# A rare cause of sudden unexpected death syndrome (SUDS) in the first year of life: endomyocardial fibroelastosis (EFE) due to two compound heterozygous *MYBPC3* mutations

**DOI:** 10.1186/s12872-021-01977-9

**Published:** 2021-04-13

**Authors:** Benno Hartung, Anne Tank, Sven Dittmann, Stefanie Ritz-Timme, Eric Schulze-Bahr

**Affiliations:** 1 Institute of Legal Medicine, University Hospital Düsseldorf, Moorenstr. 5, 40225 Düsseldorf, Germany; 2 Department of Cardiovascular Medicine, Institute for Genetics of Heart Diseases, University Hospital Münster, 48129 Münster, Germany

**Keywords:** Sudden unexpected death, MYBPC3, Compound heterozygosity, Endomyocardial fibroelastosis, Molecular autopsy

## Abstract

**Background:**

Autopsies regularly aim to clarify the cause of death; however, relatives may directly benefit from autopsy results in the setting of heritable traits (“mortui vivos docent”).

**Case presentation:**

A case of a sudden unexpected cardiac death of a 5.5-months-old child is presented. Autopsy and thorough postmortem cardiac examinations revealed a massively enlarged heart with endomyocardial fibroelastosis. Postmortem molecular testing (molecular autopsy) revealed an unusual combination of two biparental *MYBPC3* gene mutations likely to underlie the cardiac abnormalities. Thus, the molecular autoptic findings also had consequences for the relatives of the deceased child and impact on further family planning.

**Conclusions:**

The presented case highlights the need for clinical autopsies including cardiac examinations and postmortem molecular testing; it also paves the way for further cascade screening of family members for cardiac disease, if a distinct genetic disorder is suspected.

## Background

Autopsies have been performed for centuries to understand the pathologies of diseases or injuries. First systematic observations were published in 1507 [1]. In 1751, the significance of a thorough clinical case history for the interpretation of autopsy results was stressed by Boerhaave [2]. Despite different framework conditions that determine the respective type of autopsy, both pathological and forensic autopsy serve to identify and to name the underlying disease or entity. Best results are often obtained when autopsy specialists and clinicians work closely together (if allowed by the investigative authority). Systematically evaluated results may sometimes even become socially relevant [3]. More commonly, pathologies are uncovered that both clarify the cause of death and bear possible implications for the relatives, e.g. siblings [4, 5].

Endocardial fibroelastosis (EFE) is primarily a disease of infants and children presenting with unexplained heart failure (previously named as ‘fetal endocarditis’) and is characterized by thickening of endocardium due to the excessive proliferation of fibrous and elastic tissue.

This report describes the unusual case of an unexpected death of a 5.5 months-old female, in which the examinations following the autopsy brought clarity about the cause and heritability of the underlying disease, which had considerable effects on the family and further family planning. A combination of two compound heterozygous mutations in the *MYBPC3* (cardiac myosin-binding protein C3) gene was finally identified.

## Case presentation

A 5.5-months-old child died in a children's intensive care unit due to acute heart failure of unclear cause after being admitted to hospital a day before with dyspnoe and suspected bronchiolitis. The blood cell count, however, had been inconspicuous. In an ultrasound examination of the heart, a massive biventricular cardiac enlargement as well as significant biventricular dysfunction had been noticed. Therefore, an urgent transfer to a specialized heart centre was planned, but, just before, the child had a cardiopulmonary resuscitation and died after 2-h of resuscitation.

Retrospectively, the child’s birth was unremarkable (at 38th week of pregnancy, Apgar score: 9/10/10, birth weight: 3,170 g, birth length: 47 cm, umbilical cord arterial pH: 7.33). Pulsoxymetric screening was completely normal (100%) and without initial need for a cardiac or fetal echocardiography. In addition, during further pediatric examinations (week 1, month 1, months 3.5; so-called “U2-U4” examinations) no obvious abnormalities were noted including the child’s weight and length development.

At 8 weeks before death (“U4” examination), the child presented with mild respiratory noise, but a thorough physical cardiopulmonary examination revealed no abnormalities or a heart murmur. At 4 weeks before death, the child did not drink well temporarily, but improved by itself.

On the day of the child’s death, the child presented with blue lips, fever (38.2 °C), and appeared pale. Subsequently, the child was immediately transferred to hospital. In the next hours, oxygen saturation fell and oxygen had to be administered due to progressive dyspnoe. Approximately 3.5 h prior to death, a cardiac echocardiography showed an enlarged left ventricle with low ejection fraction. Shortly hereafter, the child had to be resuscitated with an unsuccessful end.

## Post mortem investigations

The autopsy showed a 5.5-months-old female of normal build (69 cm; 6360 g). There were no pathologic findings apart from the heart.

### Cardiac autopsy

Macromorphological findings were:

- Massively enlarged heart with an organ weight of 134 g (cause of death; 2% of body weight).
- Spherical expansion of the left ventricle (LV) with biventricular wall thickening (LV: 1.4 cm; RV: 0.4 cm).
- Endocardial fibrosis including near endocardium located muscular fibrosis.

Large vessels and heart valves were without pathological findings, i.e. no accompanying congenital heart disease.

### Cardiac histopathology

**Fig. 1:**
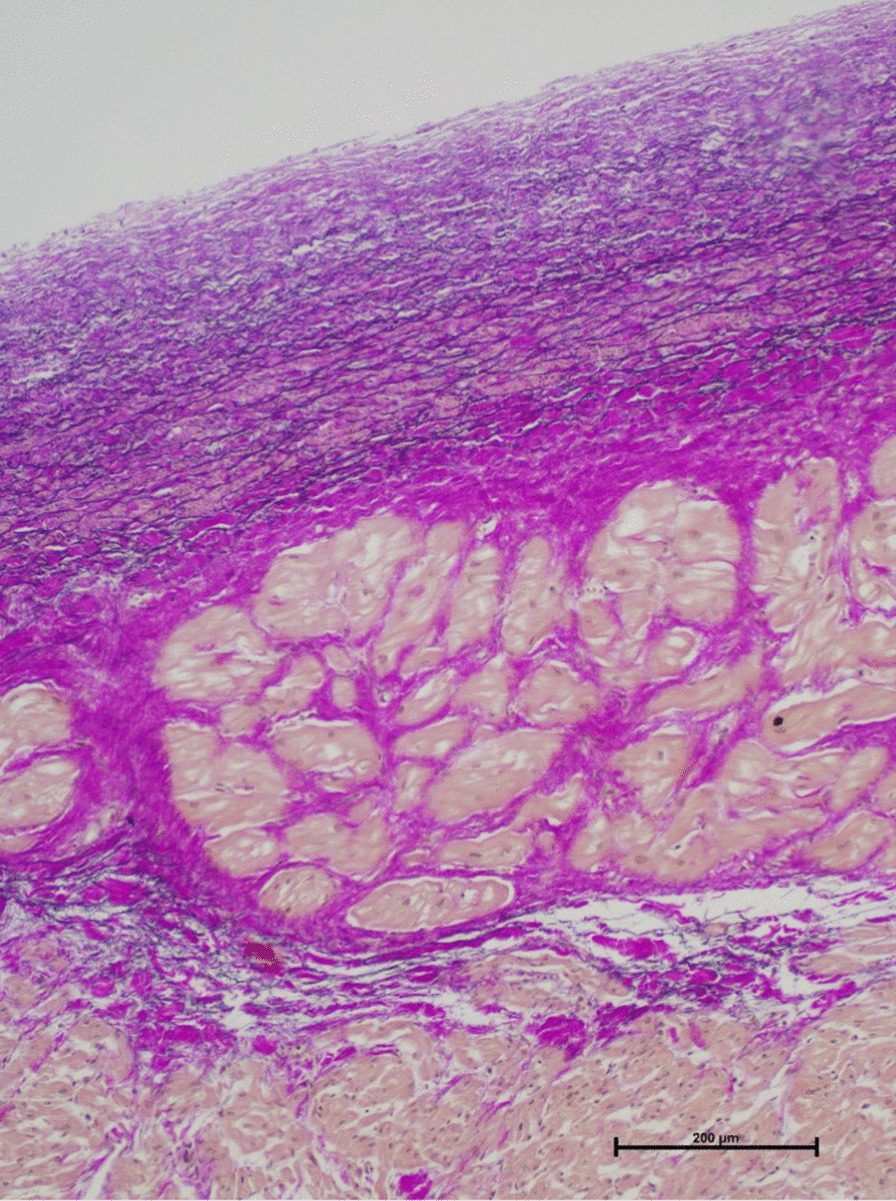
EFE of the left ventricle (Elastica-van Gieson stain, × 10) with pronounced thickening of the endocardium and, in particular, subendocardially localized fibrotic areas in the myocardial tissue

**Fig. 2:**
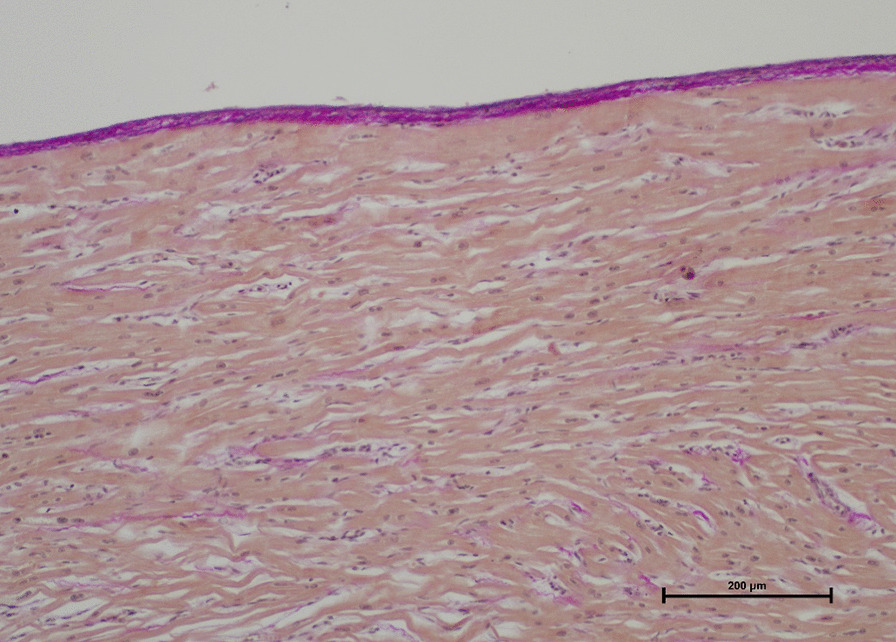
Unremarkful endocardium and myocardium of the right ventricle (Elastica-van Gieson stain, × 10)

Histopathological examinations (stains: H&E, Elastica-van Gieson, naphthol AS-D chloroacetate, CD3, CD68) revealed pronounced thickening of the endocardium and, in particular, subendocardially localized fibrotic areas in the myocardial tissue of the left ventricle. There were no signs of myocarditis, myocardial necrosis or storage disorders (Fig. 1). The RV showed a slighter endomyocardial fibroelastosis than the LV, with partially unremarkable locations (Fig. 2). This led to the diagnosis of global endomyocardial fibroelastosis (EFE).

### Postmortem molecular analysis and family cascade screening

#### Deceased child

Two heterozygous mutations in the cardiac myosin binding protein C gene (*MYBPC3*), a thick filament-associated protein located in the cross-bridge region for cardiac muscle contraction, were identified after molecular autopsy:

- a non-synonymous variant (c.592G > T, p.(Asp198Tyr); unpublished and also unknown in gnomAD (> 123,000 individuals); highly pathogenic (86% of programs) in VarCards in silico pathogenicity prediction; ACMG classification: class 4 variant (PM2, PP2, PP3, PP4, PM5_P); and
- a nonsense variant (c.2373dup, p. (Trp792Val fs*41); published loss-of-function variant, frequency in gnomAD: 0.0017% (ALL); 0.0043% (European (non-Finnish)), ACMG class 5 variant (PVS1, PS3, PS4, PP1_S, PM2, PP4).

Heterozygous mutations in *MYBPC3* primarily cause a major form of autosomal dominant hypertrophic (obstructive) cardiomyopathy (HCM; subform CMH4). Biallelic mutations are often with an early onset of HCM and severe heart failure associated (PMID: 25335496); thus, in the present case (early and severe onset of EFE with HCM) we suspected a biparental origin and performed a family cascade screening.

#### Mother of the deceased child:

Heterozygous mutation carrier of the loss-of-function *MYBPC3* mutation (c.2373dup, p. Trp792Val fs*41).

#### Father of the deceased child

Heterozygous mutation carrier of the non-synonymous *MYBPC3* mutation (c.592G > T, p. Asp198Tyr).

#### Sister of the deceased child (3.8 years-old on examination):

Heterozygous mutation carrier of the maternal mutation in *MYBPC3* (c.2373dup, p. Trp792Val fs*41).

#### Clinical examination of the family

For carriers of the *MYBPC3* mutations, regular cardiologic examinations and a genetic counselling were recommended (both parents, with sister). Mother, father and sister were completely asymptomatic and also presented a normal cardiac examination including ECG, exercise ECG and transthoracic echocardiography (no EFE or LV hypertrophy). Thus, all family members were currently without a clinical phenotype (non-penetrance), but will be followed every 3–5 years by regular cardiologic examination for development of HCM. Future family planning might be accompanied by prenatal diagnostics and detection of *MYBPC3* mutations, since there is a 25% risk for inheritance of both *MYBPC3* mutations and EFE recurrence.

## Discussion and conclusion

Fetal cardiomyopathies are a very rare disease in fetuses with a very poor outcome in the neonate. Only isolated case reports and small case series were reported, mainly presenting as dilated cardiomyopathy (DCM) with dilatation of either or both ventricles and impaired ventricular function or severe hypertrophic cardiomyopathy (HCM), as in the present case. Cardiomyopathies can be isolated or associated with other cardiac and non-cardiac malformations. Etiologically primary fetal HCM is a heterogeneous condition that can be evolutive, mainly after birth, in particular in presence of EFE.

The landmark finding established during the autopsy was a massively enlarged and hypertrophic heart muscle, consistent with endstage heart failure and HCM. In young children, the heart weight should not exceed 5‰ of the body weight (also at the age of nearly 6 months) [6], which would have been less than 32 g in the present case (here: 134 g, > fourfold).

Subsequent, histopathological evaluations revealed EFE, a primary and very rare (1: 100,000 in children) type of restrictive cardiomyopathy [7], which is said to be the most common type of restrictive cardiomyopathy [8]. Typically, the apical endocardium is affected [9] and atrioventricular valve regurgitation is common [8]. A possible familial occurrence and mostly recessive inheritance of EFE has been reported in a few reports so far. However, in many EFE cases it has been difficult to determine an environmental (e.g., parainfectious) or genetic cause or a contribution of both [8, 10]. Mostly, EFE is biventricular (50%), in 40% affecting only the LV or in 10% the RV.

The unusual autoptic findings prompted the autopsy examiners to ask the attending police officer to inform the child’s parents about a potentially heritable cardiovascular disorder and the opportunity to perform a molecular autopsy.

Finally, postmortem molecular testing (molecular autopsy) was informative and detected a quite uncommon combination and recessive inheritance of two heterozygous *MYBPC3* gene mutations. Both variants are predicted to cause a loss-of-function. The encoded protein is essential for sarcomere organization and maintenance of cardiac function; it is arrayed transversely in sarcomere A-bands and binds myosin heavy chain in thick filaments and titin (188,840) in elastic filaments. Compound heterozygous mutations in *MYBPC3* are extremely rare and currently, there are less than 30 reports. The fact that heterozygous mutation carriers (e.g., parents) with typical loss-of-function mutations did not show any signs of cardiac hypertrophy, might simply reflect an age-dependent disease penetrance. There are > 1000, various reported *MYBPC3* variants (e.g. missense/nonsense, splicing, deletions) for HCM in HGMD (Human Gene Mutation Database, accessed 02/2021). So far, a few numbers of founder mutations with a higher prevalence are known in Europe, but also in Japan and China [11]. Compound heterozygous mutations are associated with a severe cardiac hypertrophy, non-compacted LV myocardium after birth and progressive and early onset of heart failure. In such cases, cardiac death due to progressive heart failure often occurred within the first year of life [12, 13].

Obviously, the postmortem genetic results implied consequences for the relatives of the deceased child and had a significant impact on further family planning. I.a. the mother of the deceased child was thus able to undergo chorionic villus analysis during her current pregnancy. Therefore, detailed evaluation of fetal and parental condition provides prognostic information for prenatal counselling and may lead to improved outcome of at least some affected pregnancies.

Concludingly, the presented case highlights the need for clinical examination and/or genetic testing of family members in autopsy findings suspicious of genetic disorders. Mutation carriers should be informed about inheritance and consequences of these variants, which can be decisive also for family planning. Further medical examinations should be recommended to the family in these cases either directly or via police officers (depending on the investigative authority), especially in cardiac causes of death [14]. Singular persons might sometimes significantly profit of such a procedure (“mortui vivos docent”) and specialists of legal medicine should be aware of this.

## Data Availability

The data that support the findings of this study are not publicly since the data were obtained in the course of a judicial death investigation. Data are however available from the authors upon reasonable request and with permission of both the parents of the deceased child and responsible public prosecutor.

## Abbreviations

ACMG: American College of Medical Genetics and Genomics
ECG: Electrocardiogram
EFE: Endomyocardial fibroelastosis
HCM: Hypertrophic cardiomyopathy
LV: Left ventricle
MYBPC3: Cardiac myosin-binding protein C 3
RV: Right ventricle
SUDS: Sudden unexpected death syndrome
